# Mechanical ventilation using non-injurious ventilation settings causes lung injury in the absence of pre-existing lung injury in healthy mice

**DOI:** 10.1186/cc7688

**Published:** 2009-01-19

**Authors:** Esther K Wolthuis, Alexander PJ Vlaar, Goda Choi, Joris JTH Roelofs, Nicole P Juffermans, Marcus J Schultz

**Affiliations:** 1 Department of Intensive Care Medicine, University of Amsterdam, Meibergdreef 9, 1105 AZ Amsterdam, The Netherlands; 2 Department of Anesthesiology, University of Amsterdam, Meibergdreef 9, 1105 AZ Amsterdam, The Netherlands; 3 Laboratory of Experimental Intensive Care and Anesthesiology (LEICA), University of Amsterdam, Meibergdreef 9, 1105 AZ Amsterdam, The Netherlands; 4 Department of Internal Medicine, University of Amsterdam, Meibergdreef 9, 1105 AZ Amsterdam, The Netherlands; 5 Department of Pathology, University of Amsterdam, Meibergdreef 9, 1105 AZ Amsterdam, The Netherlands; 6 HERMES Critical Care Group, Amsterdam, The Netherlands

## Abstract

**Introduction:**

Mechanical ventilation (MV) may cause ventilator-induced lung injury (VILI). Present models of VILI use exceptionally large tidal volumes, causing gross lung injury and haemodynamic shock. In addition, animals are ventilated for a relative short period of time and only after a 'priming' pulmonary insult. Finally, it is uncertain whether metabolic acidosis, which frequently develops in models of VILI, should be prevented. To study VILI in healthy mice, the authors used a MV model with clinically relevant ventilator settings, avoiding massive damage of lung structures and shock, and preventing metabolic acidosis.

**Methods:**

Healthy C57Bl/6 mice (n = 66) or BALB/c mice (n = 66) were ventilated (tidal volume = 7.5 ml/kg or 15 ml/kg; positive end-expiratory pressure = 2 cmH_2_O; fraction of inspired oxygen = 0.5) for five hours. Normal saline or sodium bicarbonate were used to correct for hypovolaemia. Lung histopathology, lung wet-to-dry ratio, bronchoalveolar lavage fluid protein content, neutrophil influx and levels of proinflammatory cytokines and coagulation factors were measured.

**Results:**

Animals remained haemodynamically stable throughout the whole experiment. Lung histopathological changes were minor, although significantly more histopathological changes were found after five hours of MV with a larger tidal volume. Lung histopathological changes were no different between the strains. In both strains and with both ventilator settings, MV caused higher wet-to-dry ratios, higher bronchoalveolar lavage fluid protein levels and more influx of neutrophils, and higher levels of proinflammatory cytokines and coagulation factors. Also, with MV higher systemic levels of cytokines were measured. All parameters were higher with larger tidal volumes. Correcting for metabolic acidosis did not alter endpoints.

**Conclusions:**

MV induces VILI, in the absence of a priming pulmonary insult and even with use of relevant (least injurious) ventilator settings. This model offers opportunities to study the pathophysiological mechanisms behind VILI and the contribution of MV to lung injury in the absence of pre-existing lung injury.

## Introduction

Mechanical ventilation (MV) may aggravate pre-existing lung injury or even cause lung injury in healthy lungs, a phenomenon frequently referred to as ventilator-induced lung injury (VILI). Present strategies at minimising VILI in critically ill patients consist of using low tidal volumes (V_T_) [1]. However, additional strategies to attenuate pulmonary inflammation may be useful to further reduce VILI. Adequate animal models are also required, to test various treatment strategies. However, existing animal models of MV have considerable disadvantages.

Most models of VILI use very high V_T _and/or inspiratory pressures that are considerably higher than those used in the clinical management of patients [2-6]. High V_T _may compromise systemic circulation, eventually leading to shock. Wilson and colleagues used an MV strategy in which mice were ventilated with a V_T _of 34.5 ml/kg for a duration of 156 minutes until mean blood pressure fell below 45 mmHg [5,6]. Consequently, duration of MV is relatively short and maybe too short to draw meaningful conclusions. In addition, most models of VILI lungs are 'primed' before starting MV [7-11]. Indeed, animals are challenged before onset of MV, for instance for lipopolysaccharide causing lung injury [7,11]. Such an approach prevents conclusions on the deleterious effects of MV in the absence of pre-existing lung injury being drawn. One final problem may be that infusion of saline solution to correct for low arterial blood pressures leads to metabolic acidosis in models of VILI [12,13], although metabolic acidosis may influence several endpoints of VILI [14,15]. It is uncertain whether metabolic acidosis should be corrected in models of VILI.

The aim of the present investigation was to set up a model of VILI in healthy animals. We chose an MV strategy that closely reflected the human setting by using clinically relevant V_T_, preventing shock and gross lung histopathological changes, and compared lower V_T _with higher V_T _with respect to several endpoints of VILI. In addition, we hypothesised preventing metabolic acidosis to affect endpoints of VILI. Therefore we compared two strategies for fluid resuscitation, using either normal saline or sodium bicarbonate.

## Materials and methods

The study was approved by the Animal Care And Use Committee of the Academic Medical Center. Animal procedures were carried out in compliance with Institutional Standards for Human Care and Use of Laboratory Animals.

### Animals

Experiments were performed with healthy male C57Bl/6 (n = 66) and BALB/c mice (n = 66) (Charles River, Someren, the Netherlands), aged 8 to 10 weeks, with weights ranging from 19 to 25 g. Two groups of control animals served either as non-ventilated controls for blood gas analysis at baseline (n = 6 for each strain) or as non-ventilated controls after five hours (n = 12 for each strain). The other animals were all mechanically ventilated with two different MV-strategies and two different fluid support strategies. Thus, five groups of animals of each mice strain were compared.

### Instrumentation and anesthesia

Throughout the experiments, rectal temperature was maintained between 36.5 and 37.5°C using a warming path. Anaesthesia was achieved with intraperitoneal injection of a mix of 100 mg/ml ketamine (Eurovet Animal Health B.V., Bladel, the Netherlands), 1 mg/ml medetomidine (Pfizer Animal Health B.V., Capelle a/d IJssel, the Netherlands) and 0.5 mg/ml atropine (Pharmachemie, Haarlem, the Netherlands; KMA). Induction of anaesthesia was performed by injecting 7.5 μl/g of induction KMA mix (consisting of 1.26 ml ketamine, 0.2 ml medetomidine and 1 ml atropine). To maintain anaesthesia, 10 μl/g of maintenance KMA mix (consisting of 0.72 ml ketamine, 0.08 ml medetomidine and 0.3 ml atropine) was given, via an intraperitoneally placed catheter every hour.

### Mechanical ventilation strategies

A Y-tube connector, 1.0 mm outer diameter and 0.6 mm inner diameter (VBM Medizintechnik GmbH, Sulz am Neckar, Germany) was surgically inserted into the trachea under general anaesthesia. Mice were placed in a supine position and connected to a ventilator (Servo 900 C, Siemens, Sweden). Simultaneously, six mice were pressure-controlled ventilated with either an inspiratory pressure of 10 cmH_2_O (resulting in V_T _of about 7.5 ml/kg; low V_T _(LV_T_)) or an inspiratory pressure of 18 cmH_2_O (resulting in V_T _of about 15 ml/kg; high V_T _(HV_T_)).

In C57Bl/6 mice, respiratory rate was set at 120 breaths/minute and 70 breaths/minute with LV_T _and HV_T_, respectively; in BALB/c mice, respiratory rate was set at 100 breaths/minute and 70 breaths/minute with LV_T _and HV_T_, respectively. Preliminary studies showed these respiratory settings resulted in normal partial pressure of arterial carbon dioxide (PaCO_2_) values after five hours of MV in the different mice strains. Positive end-expiratory pressure (PEEP) was set at 2 cmH_2_O with both MV strategies. The fraction of inspired oxygen was kept at 0.5 throughout the experiment. The inspiration to expiration ratio was kept at 1:1 throughout the experiment.

### Fluid support strategies

Mice received an intraperitoneal bolus of 1 ml normal saline one hour before the start of MV, followed by 0.2 ml normal saline (sodium chloride (NaCl) 0.9%) or 0.2 ml sodium bicarbonate (containing 200 mM sodium and bicarbonate) administered via the intraperitoneal catheter every 30 minutes. Preliminary studies showed this fluid strategy to adequately compensate for insensible and observed fluid loss, and to keep the animals haemodynamically stable.

### Haemodynamic and ventilatory monitoring

Systolic blood pressure and heart rate were non-invasively monitored using a murine tail-cuff system (ADInstruments, Spenbach, Germany). Blood pressure and pulse were measured directly after the start of MV, after 2.5 hours and 5 hours of MV. The data were recorded on a data acquisition system (PowerLab/4SP, ADInstruments, Spenbach, Germany). An average systolic blood pressure and heart rate were taken from three consecutive measurements.

V_T _was checked hourly with a specially designed Fleisch-tube connected to the body-plethysmograph. The flow signal was integrated from a differential pressure transducer and data were recorded and digitised online using a 16-channel data acquisition program (ATCODAS, Dataq Instruments Inc, Akron, OH) and stored on a computer for post acquisition off-line analysis. A minimum of five consecutive breaths were selected for analysis of the digitised V_T _signals.

### Study groups

Non-ventilated control mice were selected for blood gas analysis at baseline (for both strains n = 6): animals were handled one week before the experiment to decrease stress activation. After induction of anaesthesia with isoflurane arterial blood was taken from the left ventricle by heart puncture within 30 seconds.

LV_T _mice receiving either normal saline (n = 12) or sodium bicarbonate (n = 12) and HV_T _mice receiving either saline (n = 12) or sodium bicarbonate (n = 12) were mechanically ventilated for five hours and then euthanased. Non-ventilated control mice (n = 12) received half the dose of induction anaesthesia, were spontaneously breathing and then euthanased after five hours.

### Measurements

The first series of mice (n = 6) were euthanased and blood was drawn from the vena cava inferior into a sterile syringe, transferred to EDTA-coated tubes and immediately placed on ice. Blood samples of two mice were pooled together. Bronchoalveolar lavage fluid (BALF) was obtained from the right lung; the left lung was used to measure the wet-to-dry ratio. In a second series of mice (n = 6), blood was sampled from the carotid artery for blood gas analysis. The lungs of these mice were used for homogenate (right lung) and histopathology (left lung).

For wet-to-dry ratios the lung was weighed and subsequently dried for three days in an oven at 65°C. The right lung was removed and snap frozen in liquid nitrogen. These frozen specimens were suspended in four volumes of sterile isotonic saline and subsequently lysed in one volume of lysis buffer (150 mM NaCl, 15 mM Tris (tris(hydroxymethyl)aminomethane), 1 mM MgCl.H_2_O, 1 mM CaCl_2_, 1% Triton X-100, 100 μg/mL pepstatin A, leupeptin and aprotinin, pH 7.4) and incubated at 4°C for 30 minutes. Homogenates were spun at 3400 rpm at 4°C for 15 minutes after which the supernatants were stored at -20°C until assayed.

BALF was obtained by instilling three times 0.5 ml aliquots of saline by a 22-gauge Abbocath–T catheter (Abbott, Sligo, Ireland) into the trachea. About 1.0 ml of BALF was retrieved per mouse and cell counts were determined using a haemacytometer (Beckman Coulter, Fullerton, CA). Subsequently, differential counts were performed on citospin preparations stained with a modified Giemsa stain, Diff-Quick (Dade Behring AG, Düdingen, Switzerland). Supernatant was stored at -80°C for meausrement of total protein level, thrombin-antithrombin complexes (TATc) and plasminogen activator inhibitor (PAI)-1.

### Lung histopathology

For histopathology lungs were fixed in 4% formalin and embedded in paraffin. Sections 4 μm in diameter were stained with H&E and analysed by a pathologist who was blinded for group identity. To score lung injury we used a modified VILI histopathology scoring system as previously described [2]. VILI was scored according to the following four items: alveolar congestion; haemorrhage; infiltration or aggregation of neutrophils in airspace or vessel wall; and thickness of the alveolar wall/hyaline membrane formation. A score of 0 represented normal lungs; 1 represented mild, less than 25% lung involvement; 2 represented moderate, 25 to 50% lung involvement; 3 represented severe, 50 to 75% lung involvement; and 4 represented very severe, more than 75% lung involvement. An overall score of VILI was obtained based on the summation of all the scores from normal or ventilated lungs (n = 12 per group).

### Assays

Total protein levels in BALF were determined using a Bradford Protein Assay Kit (OZ Biosciences, Marseille, France) according to the manufacturers' instructions with BSA as standard. Cytokine levels in blood lung homogenates were measured by ELISA according to the manufacturer's instructions. Tumour necrosis factor (TNF) α, interleukin (IL) 6, macrophage inflammatory protein (MIP) 2 and keratinocyte-derived chemokine (KC) assays were all obtained from R&D Systems (Abingdon, UK). TATc levels in BALF were measured with a mouse specific ELISA as previously described [16]. Levels of PAI-1 were measured by means of a commercially available ELISA (Kordia, Leiden, the Netherlands).

### Statistical analysis

All data in the results are expressed as mean ± standard deviation or median ± interquartile range (IQR), where appropriate. To detect differences between groups the Dunnett method or Mann Whitney U test, in conjunction with two-way analysis of variance was performed. Haemodynamics were measured in 12 animals, all other measurements were performed in six animals. A p value of less than 0.05 was considered significantly. All statistical analyses were carried out using SPSS 12.0.2 (SPSS, Chicago, IL).

## Results

### Haemodynamic and ventilatory monitoring

All animals survived five hours of MV after which they were euthanased; control animals survived anaesthesia and were also euthanased after five hours. The systolic blood pressure and heart rate remained stable in all animals for the entire duration of the experiment, with no differences noted between mice strains, MV strategies and fluid strategies. Although blood gas analysis from LV_T _mice and HV_T _mice using normal saline revealed metabolic acidosis after five hours of MV (in C57Bl/6 mice pH with LV_T _= 7.17 ± 0.07 and pH with HV_T _= 7.23 ± 0.06, and in BALB/c mice pH with LV_T _= 7.22 ± 0.04 and pH with HV_T _= 7.11 ± 0.07, Tables 1 and 2) with the use of sodium bicarbonate metabolic acidosis was prevented (in C57Bl/6 mice pH with LV_T _= 7.41 ± 0.07 and pH with HV_T _= 7.49 ± 0.02, and in BALB/c mice pH with LV_T _= 7.42 ± 0.05 and pH with HV_T _= 7.37 ± 0.08). Arterial oxygenation in C57Bl/6 mice was significantly higher in HV_T_-mice as compared with LV_T_-mice (205 ± 33 *vs. *141 ± 22 mmHg, p < 0.001). No differences regarding oxygenation were found between MV-groups in BALB/c mice (partial pressure of arterial oxygen (PaO_2_) for HV_T _= 167 ± 50 and PaO_2 _for LV_T _= 181 ± 42 mmHg).

**Table 1:** Arterial blood gas analysis in C57Bl/6 mice.

	**Control**	**Low V**_T_		**High V**_T_	
	
		NaCl	NaHCO_3_	NaCl	NaHCO_3_
	
pH	7.42 (0.04)	7.17 (0.07)‡	7.41 (0.07)	7.23 (0.06)‡	7.49 (0.02)
PaCO_2 _(mmHg)	34.4 (32.2 to 38.3)	50.1 (36.7 to 59.6)	45.0 (38.6 to 50.0)	33.7 (32.1 to 34.0)	31.0 (27.6 to 34.4)
PaO_2 _(mmHg)		133 (15)	148 (28)	186 (45)	223 (20)
HCO_3_^- ^(mmol/l)	21.4 (21.1 to 24.1)	16.6 (15.2 to 18.9)	28.0 (26.1 to 30.0)	14.6 (13.3 to 15.6)	24.9 (21.3 to 25.5)
BE	-1.3 (-2.3 to -0.5)	-11.7 (-12.5 to -10.2)	4.1 (1.3 to 6.0)	-12.8 (-13.4 to -10.1)	2.3 (-0.4 to 2.9)

Data are mean (SD) or median [IQR]; Control = spontaneously breathing mice; Low V_T _= mice ventilated for five hours with a V_T _of 7.5 ml/kg; High V_T _= mice ventilated for five hours with a V_T _of 15 ml/kg. n = 6 per group. *p < 0.05; ‡p < 0.001 *vs*. control mice.

PaCO_2 _= partical pressure of arterial carbon dioxide; PaO_2 _= partical pressure of arterial oxygen; BE = base excess.

**Table 2:** Arterial blood gas analysis in BALB/c mice.

	**Control**	**Low V**_T_		**High V**_T_	
	
		NaCl	NaHCO_3_	NaCl	NaHCO_3_
	
PH	7.34 (0.05)	7.22 (0.04)*	7.42 (0.05)	7.11 (0.07)‡	7.37 (0.08)
PaCO_2 _(mmHg)	39.3 (31.6 to 51.3)	35.7 (31.1 to 39.5)	41.2 (35.3 to 43.6)	40.8 (37.0 to 55.6)	44.3 (36.1 to 51.7)
PaO_2 _(mmHg)		193 (36)	168 (48)	173 (51)	161 (50)
HCO_3_^- ^(mmol/l)	21.1 (17.9 to 24.1)	14.4 (12.9 to 15.2)	25.2 (23.6 to 25.9)	13.5 (12.1 to 14.7)	24.5 (22.7 to 25.4)
BE	-3.9 (-6.2 to -2.5)	-12.3 (-13.6 to -11.8)	0.15 (-1.1 to 2.2)	-15.9 (-16.7 to -14.8)	-0.7 (-2.4 to -0.1)

Data are mean (SD) or median (IQR); Control = spontaneously breathing mice; Low V_T _= mice ventilated for five hours with a V_T _of 7.5 ml/kg; High V_T _= mice ventilated for five hours with a V_T _of 15 ml/kg. n = 6 per group. ‡p < 0.001 *vs*. control mice. PaCO_2 _= partical pressure of arterial carbon dioxide; PaO_2 _= partical pressure of arterial oxygen; BE = base excess.

### Lung histopathology scores

**Figure 1:**
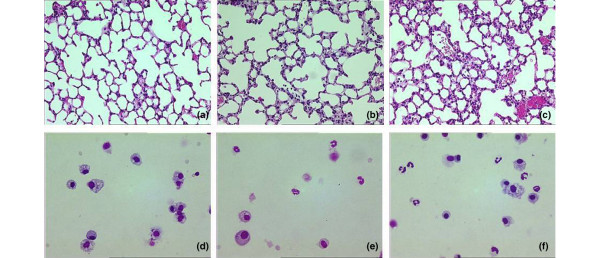
**Histological specimens from the lungs of spontaneously breathing mice and mice ventilated with low/high tidal volumes**. (a to c) Images of histological specimens from the lungs of spontaneously breathing C57Bl/6 mice (control) or ventilated with low tidal volumes (LV_T_) and high V_T _(HV_T_) for five hours. H&E stain; magnification 200×. **(a) **Control mice; **(b) **LV_T _mice; **(c) **HV_T _mice. (d to e) Images of citospin preparations of BALF of C57Bl/6 mice stained with Diff-Quick. **(d) **control mice; **(e) **LV_T _mice; **(f) **HV_T _mice.

The histopathological changes were minor (Figure 1 and Table 3). For both mice strains the lung histopathology score was higher in HV_T _mice as compared with controls. However, no differences were noted between mice strains, MV strategies and fluid strategies.

**Table 3:** Cell counts in lung lavage fluid and histopathological examination of lung tissue of C57Bl/6 mice.

	Control	LV_T_	HV_T_
Total cells (× 10^4^/ml BALF)	44 (30 to 45)	23 (14 to 221)	14 (10 to 20)
Neutrophils (× 10^4^/ml BALF)	0.13 (0.0 to 0.73)	1.9 (1.2 to 2.8)	4.5 (3.9 to 12.7)*
VILI–score	0.0 (0.0 to 0.5)	1.0 (0.0 to 3.0)	2.0 (1.0 to 4.5)*

Data are presented as median (IQR). Control = spontaneously breathing mice, LV_T _= low tidal volumes, HV_T _= high tidal volumes, BALF = broncho-alveolar lavage fluid, VILI = ventilator-induced lung injury. n = 6 per group. *p < 0.05 *vs*. control.

### Wet-to-dry ratios, BALF-protein content and neutrophil influx

In C57Bl/6 mice lung wet-to-dry ratios were significantly higher with both MV strategies compared with controls (LV_T _mice = 4.8 ± 0.3 and HV_T _mice = 5.3 ± 0.5, as compared with control mice = 4.2 ± 0.2; p < 0.01). Wet-to-dry ratios in HV_T _mice were also significantly higher as compared with LV_T _mice (p = .009). For BALB/c mice higher lung wet to dry ratios were found in HV_T _mice (5.6 ± 0.6 as compared with 4.6 ± 0.4 in LV_T _mice (p < 0.001) and 4.5 ± 0.2 in control mice (p < 0.001), respectively). No significant differences were found between LV_T _mice and control mice.

**Figure 2:**
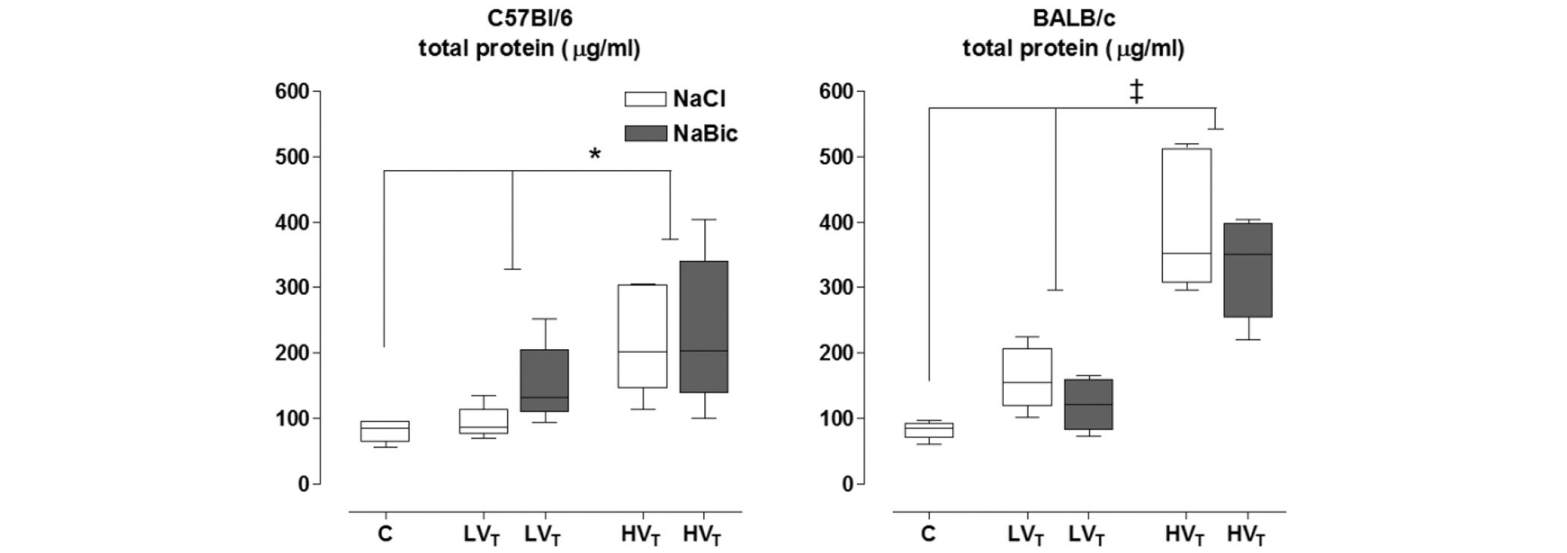
**Total protein level in control mice and mice ventilated with low/high tidal volumes**. Total protein level in control mice, and in mice ventilated with low tidal volumes (LV_T_) and high V_T _(HV_T_) for five hours. Two fluid strategies (normal saline (white boxes) and sodium bicarbonate (grey boxes)) were compared. Data represent median and interquartile range of six mice. *p < 0.05 (HV_T _*vs*. LV_T_); ‡p < 0.001 (HV_T _*vs*. LV_T_).

Total BALF protein levels in C57Bl/6 were significantly higher in HV_T _mice as compared with LV_T _mice (p = .012) and control mice (p = .008; Figure 2). No significant difference was found between LV_T _mice and control mice. In BALB/c mice, total BALF protein levels were significantly higher in HV_T _mice as compared with LV_T _mice and control mice (p < .001). No significant difference was found between LV_T _mice and control mice.

The numbers of neutrophils in BALF were significantly higher in HV_T _mice as compared with control mice in both mice strains (Figure 1 and Table 3). Neutrophil counts in BALF from HV_T _mice did not differ from LV_T_mice.

### Pulmonary and plasma cytokine levels

**Figure 3:**
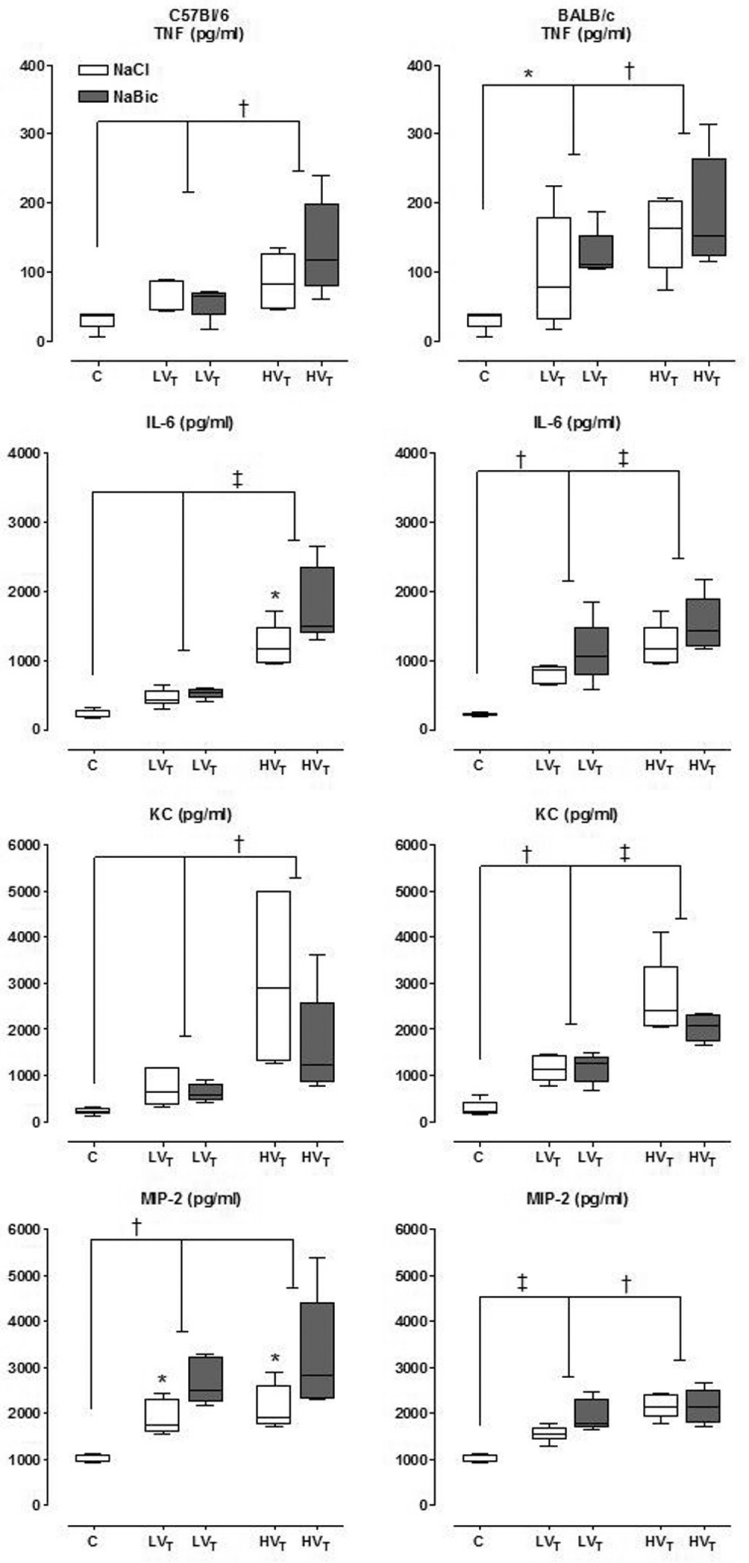
**Pulmonary levels of tumour necrosis factor (TNF)-α, interleukin (IL)-6, keratincyte-derived cytokine (KC) and macrophage inflammatory protein (MIP)-2 in lung tissue homogenate**. Pulmonary levels of TNF-α, IL-6, KC and MIP-2 and in lung tissue homogenate in control mice, and in mice ventilated with low tidal volumes (LV_T_) and high V_T _(HV_T_) for five hours. Two fluid strategies (normal saline (white boxes) and sodium bicarbonate (grey boxes)) were compared. Data represent median and interquartile range of six mice. *p < 0.05 (LV_T _*vs*. control or sodium bicarbonate *vs*. saline, IL-6 and MIP-2 in C57Bl/6 mice); †p < 0.01 (HV_T _*vs*. LV_T _or LV_T _*vs*. control); ‡p < 0.001 (HV_T _*vs*. LV_T _or LV_T _*vs*. control).

In the HV_T _group of both mice strains, higher pulmonary levels of TNF-α were found as compared with the LV_T _group (p < 0.05) and control group (p≤ 0.001; Figure 3). In BALBc mice only, pulmonary levels of TNF-α in LV_T _mice were higher as compared with control mice (p = 0.018). Pulmonary levels of IL-6 in the HV_T _group of both mice strain were higher as compared with the LV_T _group and control group. Only for BALBc mice a significant difference between LV_T _mice and control mice were found. For pulmonary levels of MIP-2 in C57Bl/6 mice higher levels were found in HV_T _mice and LV_T _mice as compared with control (p = 0.001). No difference was found between LV_T _mice and HV_T _mice in this mice strain. In BALBc mice, higher pulmonary levels of MIP-2 in the HV_T _group were found as compared with the LV_T _group and control group, with also a significant difference between HV_T _mice and LV_T _mice. In both mice strain higher pulmonary levels of KC were found in the HV_T _group as compared with the LV_T _group and control group (p = 0.001). Only in BALBc mice, there was also a significant difference between LV_T _mice and control mice.

**Figure 4:**
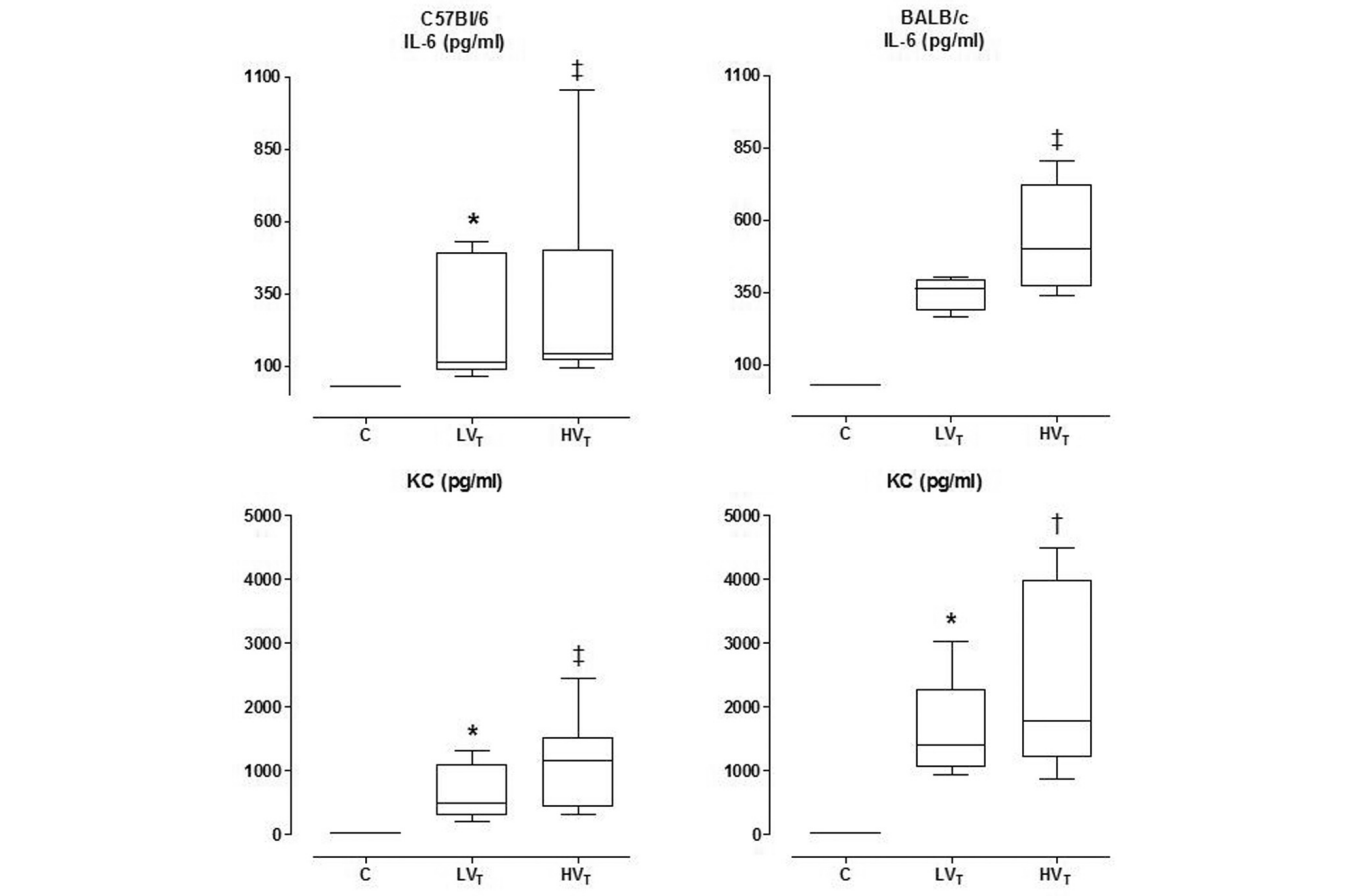
**Plasma levels of interleukin (IL)-6 and keratinocyte-derived chemokine (KC)**. Plasma levels of IL-6 and KC in control mice, and in mice ventilated with low tidal volumes (LV_T_) and high V_T _(HV_T_) for five hours. Data of the two fluid strategies are pooled. Data represent median and interquartile range of six mice. Levels of IL-6 and KC in control mice were below the detection limit of the assay. *p < 0.05 *vs*. control; †p < 0.01 *vs*. LV_T_; ‡p < 0.001 *vs. *LV_T_.

Plasma levels of IL-6 and KC were elevated in the both ventilation groups, with higher levels in the HV_T _group (Figure 4). Plasma levels of TNF-α and MIP-2 were below the detection limit of the assay (data not shown).

### Pulmonary coagulopathy

**Figure 5:**
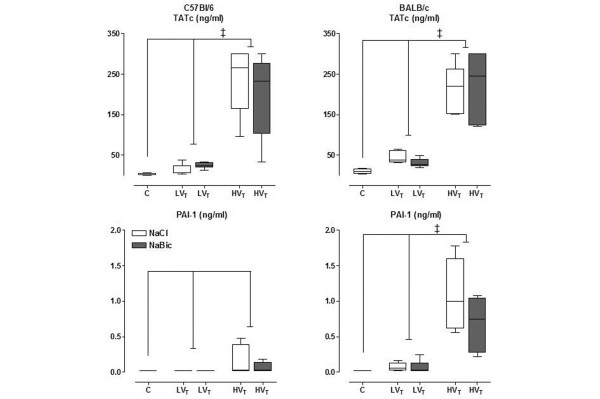
**Thrombin-antithrombin complexes (TATc) levels and plasminogen activator inhibitor (PAI)-1 levels in bronchoalveolar lavage fluid**. TATc levels and PAI-1 levels in bronchoalveolar lavage fluid in control mice, and in mice ventilated with low tidal volumes (LV_T_) and high V_T_(HV_T_) for five hours. Two fluid strategies (normal saline (white boxes) and sodium bicarbonate (grey boxes)) were compared. Data represent median and interquartile range of six mice. ‡p < 0.001 (HV_T _*vs*. LV_T_).

TATc levels in BALF were significantly higher in HV_T _mice in both mice strain as compared with LV_T _mice and control (p < 0.001; Figure 5). No significant difference was found between LV_T _mice and control mice in both mice strain. Levels of PAI-1 were not significantly different in C57Bl/6 mice. BALB/c mice did show increased PAI-1 levels in the HV_T_group as compared with the LV_T _group and control group (p < 0.001). No differences were found between LV_T _mice and control mice.

### Lung injury with different fluid support strategies

The different fluid support strategies showed no difference in endpoint of VILI, except for pulmonary MIP-2 and IL-6 levels in C57Bl/6 mice. MIP-2 levels were significantly higher in HV_T _mice and LV_T _mice that received sodium bicarbonate as compared with mice that received normal saline (p < 0.01; Figure 3). Pulmonary IL-6 levels were significantly higher in HV_T _mice receiving sodium bicarbonate as compared with mice receiving normal saline (p = 0.026).

## Discussion

We here show MV to cause VILI in healthy lungs (i.e. in the absence of a 'priming' lung insult). VILI did not only develop in animals ventilated with HV_T _but also in animals ventilated with LV_T_, although to a lesser extent. We chose an MV strategy that closely reflects the human setting by using clinically relevant (i.e. physiological) V_T_, preventing shock and gross lung histopathological changes. Although we hypothesised that preventing metabolic acidosis would affect the several endpoints of VILI, we showed that correction of the acid-base balance did not affect VILI.

We developed and tested a model of VILI in two commonly used mice strains using clinically relevant V_T _and preventing hypovolaemia with fluid support. By using a clinically relevant V_T _and fluid support we prevented shock. By using sodium bicarbonate instead of normal saline, metabolic acidosis was prevented. We developed a model that enhances translation of results into clinical practice and/or future studies. To our best knowledge, this is one of the first studies that compares more physiological V_T _then previously used in healthy lungs of mice.

Our model has several limitations. First, V_T _in HV_T _mice are still quite large (about 15 ml/kg). Although lung-protective ventilation with the use of LV_T _is underused in patients with acute lung injury (ALI)/adult respiratory distress syndrome (ARDS) [17] and patients at risk for ALI/ARDS [18], in the clinical arena V_T _have declined gradually over the past 10 years [19,20]. However, V_T _of as large as 15 ml/kg are still reported to be used [21,22]. Therefore our comparison may still reveal relevant information on lung injury caused by MV.

Second, LV_T _ventilation can promote development of atelectasis. This may, in part, explain the lower oxygenation levels with use of LV_T _in our experiments. It was recently demonstrated that periodic recruitment with relatively frequent deep inflations during ventilation with LV_T _can improve oxygenation, ventilation and lung mechanical function with no evidence of lung injury by two hours in mechanically ventilated mice [23]. Therefore, lung injury seen in our LV_T _mice could be caused by atelectotrauma.

Third, our non-ventilated control animals were not sham operated, did not receive fluid resuscitation and were breathing room air as opposed to our ventilated animals. It can be suggested that the invasive surgical procedure has an influence on the inflammatory reaction by entering endotoxins and/or bacteria into the circulation. MV in combination with prolonged exposure to hyperoxia (> 95% of oxygen) augmented lung injury [24]. However, lung injury caused by 50% of oxygen, as used in our ventilated mice, has not been previously reported.

Fourth, in accordance with previous models of murine ventilation, we did not use moisture breathing gas. The problem is that drops will obstruct the inspiratory tubing. We do realise that this is a limitation of our and previous models of murine ventilation.

VILI was clearly present with the use of HV_T _after five hours of MV. For most of our endpoints of VILI significant differences were found between HV_T _mice and LV_T _mice. Of more interest, with LV_T _VILI also developed. This finding is in accordance with a previous report, where low V_T _(8 ml/kg) for four hours in mice resulted in a reversible inflammatory reaction, while preserving tissue integrity [25]. On the other hand, Altemeier and colleagues demonstrated that MV with tidal volumes of 10 ml/kg for six hours did not cause significant cytokine expression [26]. In the study of Altemeier and colleagues, cytokines were measured in the BALF, while in our study and in the study of Vaneker and colleagues cytokines were measured in lung homogenate. Maybe cytokines were still in the sub-epithelium and did not migrate further into the alveoli. Thus, even the use of LV_T _could be considered to be potentially harmful, at least in a murine setting. In disagreement with some reports that did not show any effect of larger V_T_in patients with non-injured lungs [21,22], several articles did display harmful effects of large V_T_. In one study on postoperative MV after cardiopulmonary bypass surgery, MV with tidal volumes of 6 ml/kg predicted bodyweight (PBW) resulted in significantly lower BALF TNF-α levels as compared with tidal volumes of 12 ml/kg PBW [27]. These results were confirmed by others, who showed that the use of large tidal volumes of 10 to 12 ml/kg resulted in an increase of bronchoalveolar lavage fluid and plasma IL-6 and IL-8 levels as compared with lower V_T _of 8 ml/kg [28]. In our study, patients ventilated with HV_T _(12 ml/kg PBW) for five hours showed upregulation of pulmonary inflammatory mediators as opposed to patients ventilated with LV_T _(6 ml/kg) [29]. Unrecognised differences in MV between mice and the human setting may be responsible for this difference.

With V_T _as used in our experiments histopathological changes were minor. In previously published studies the VILI score was about 2 in the low V_T _or low pressure group and about 7 in the high V_T _or high pressure group [2,30]. Worth mentioning is that V_T _or pressures used in the high V_T _group in these former studies were about twice as high as in our study protocol. In a previously mentioned study in which C57Bl/6 mice were ventilated for four hours with V_T _of 8 ml/kg, electron microscopy revealed intact epithelial cell and basement membranes with sporadically minimal signs of partial endothelial detachment [25].

Although it is well known that acid-base parameters are reliable indicators of the general condition of the animal, these parameters are not or only partly assessed in previous murine models of MV [2,9,26,31]. Acid-base balance in spontaneously breathing mice are mainly under isoflurane anaesthesia [12] and reported values on pH are rather acidotic [32]. It has been suggested that mice have a considerably lower alveolar and arterial PCO_2 _than other mammals (PaCO_2 _ranging from 33 to 41 mmHg). However, instrumentation of animals cannot be completely excluded as causative [33]. Here we show normal values for pH and PaCO_2 _in C57BL/6 mice and BALB/c mice after brief anaesthesia. Our animals developed metabolic acidosis when normal saline was used. Metabolic acidosis in mice can be induced by isoflurane anaesthesia and/or saline administration [12,13]. However we can not totally exclude that metabolic acidosis was not caused by some haemodynamic impairment, although blood pressure measured during five hours of MV was stable. Probably the effects of anaesthetics during five hour of MV are more impressive in terms of fluid losses. For this reason we choose a fluid resuscitation regimen of 0.2 ml for 30 minutes intraperitoneally. In the present study we only found subtle differences in endpoints of VILI between the two fluid therapies. Nevertheless, we favour the use of sodium bicarbonate instead of normal saline as fluid support therapy to prevent metabolic acidosis, because severe acidosis may influence unmeasured endpoints of VILI.

We found higher plasma levels of KC and IL-6 as compared with control mice and levels were higher in HV_T _mice. This finding is in accordance with data from human studies. Indeed, in patients with ALI/ARDS a lung protective MV strategy using LV_T _and sufficient PEEP levels resulted in significantly lower systemic inflammatory mediators as compared with ALI/ARDS patients ventilated with a more conventional MV strategy, using HV_T _[34].

We chose an one-hit model instead of a two-hit model to avoid the interference of an additional source of inflammation. Whether MV *per se *initiates pulmonary inflammation in patients with non-injured lungs is still unclear, although we have shown that a lung protective MV strategy (V_T _of 6 ml/kg PBW and 10 cmH_2_O PEEP) attenuates pulmonary coagulation caused by a more conventional MV strategy (V_T _of 12 ml/kg and no PEEP) [35]. In addition, MV with lower V_T _and PEEP attenuated the increase of pulmonary levels of IL-8, myeloperoxidase and elastase as seen with higher V_T _and no PEEP [29]. The inflammatory changes observed in healthy lungs are merely physiological adaptations to the artificial process of MV. Our model offers opportunities to study the pathophysiological mechanisms behind VILI and the contribution of MV to the 'multiple-hit' concept.

Several studies suggest pulmonary coagulopathy is also a feature of VILI. Indeed, we have shown that MV using high V_T _resulted in increased alveolar thrombin generation [35]. It is likely that the alveolar epithelium can initiate intra-alveolar coagulation by expressing active tissue factor [36]. Recently, we also showed MV with high V_T _to attenuate fibrinolysis in rats, in part via upregulation of PAI-1 [7,37]. These results are in line with results from the present study. Of note, use of LV_T _also resulted in profound procoagulant changes, underlining the fact that even a lung protective MV strategy to induce VILI in healthy mice.

## Conclusions

In this model of VILI in two commonly used mice strains we show physiological V_T _to induce VILI in healthy mice. Lung injury was found with both V_T _used in our experiments (i.e. also with LV_T _VILI developed). This model offers opportunities to study the pathophysiological mechanisms behind VILI and the contribution of MV to lung injury in the absence of pre-existing lung injury.

## Key messages

• MV induces VILI in mice, in the absence of a priming pulmonary insult, with use of relevant ventilator settings.

• By using sodium bicarbonate instead of normal saline metabolic acidosis was prevented.

• Endpoints of VILI were not influenced by metabolic acidosis.

## Abbreviations

BALF: broncho-alveolar lavage fluid; ELISA: enzyme-linked immunosorbent assay; H&E: haematoxylin & eosin; HV_T_: High tidal volume; IL: interleukin; IQR: interquartile range; KC: keratinocyte-derived chemokine; LV_T_: low tidal volume; MIP: macrophage inflammatory protein; MV: mechanical ventilation; PaCO_2_: partial pressure of arterial carbon dioxide; PAI: plasminogen activator inhibitor; PaO_2_: Partial pressure of arterial oxygen; PBW: predicted bodyweight; PEEP: positive end-expiratory pressure; SD: standard deviation; TATc: thrombin-antithrombin complexes; TNF: tumour necrosis factor; VILI: ventilator-induced lung injury; V_T_: tidal volume.

## Competing interests

The authors declare that they have no competing interests.

## Authors' contributions

EW performed the experimental work, interpreted the results and drafted the manuscript. AV and GC performed the experimental work and were responsible for critical review of the manuscript. JR performed part of the experimental work. NJ participated in drafting and reviewing the manuscript. MS participated in study design, interpretation of the results and drafting the manuscript. All authors read and approved the final manuscript.
